# Illinois State Veterinary Medical Association

**Published:** 1891-04

**Authors:** J. F. Pease

**Affiliations:** Recording Secretary


### SOCIETY PROCEEDINGS.

---

*Illinois State Veterinary Medical Association.*—The Illinois State Veterinary Medical Association held its regular semi-annual meeting, Feb. 25, at the Windsor Hotel, Bloomington. The meeting was called to order at 10 A. M., by President S. S. Baker, of Chicago, a quorum responded to roll call. The following gentlemen were in attendance during this meeting: Drs. Jas. Addison, S. S. Baker, J. W. Harwood, C. E. Hollingsworth, J. F. Ryan, C. E. Sayre, Jno. Scott, R. J. Withers, W. L. Williams, Jas. McClintock, Jas. T. Nathers, J. F. Pease, H. Thompson, A. G. Alverson, N. J. Stringer, M. Wilson and G. L. Crocker, (new member).

Several members elect were present but did not qualify.

The minutes were read and approved, after which correspondence was heard from Prof. McIntosh regretting absence, and from Drs. Walker and Schoenleber regretting absence and sending their papers.

The corresponding secretary also reported correspondence with the Secretary of the U. S. Veterinary Medical Association in regard to its next meeting. The business report was read and placed on file. The committee on reprinting the Constitution and By-laws reported a rate of $15.00 per hundred. Better figures were given by members outside the committee. On motion the subject was deferred and the committee held over.

The following new members were proposed: Drs. T. J. Gunning (Chicago '90), Neponset; Jno. Miller (Montreal '88), Dekalb; J. W. Parkinson (Chicago '90), El Paso; and N. P. Whitmore (Chicago '90), Gardner.

On motion the rules were suspended and they were unanimously elected to membership by acclamation.

On motion by Dr. Williams, seconded by Dr. Hollingsworth, the order of business was suspended and a committee appointed to revise the Constitution and By-laws and report at this meeting. The chair appointed Drs. Williams, Withers and Hollingsworth. Bills were then read to the society and ordered paid.

Dr. Wilson, of Nundota, then presented a well written paper on "Diseased Meats as Food for Human Consumption." Later researches have proven the indentity of certain diseases of men and of animals. The Jews recognized the connection between diseased flesh and man's health. Their code of inspections clearly describes diseases well known now, and well described and named, and *Tuberculosis* is one of these. The Rabbi's were the medical men of the ancient Jews.

Everyone now concedes bovine and human tuberculosis to be identical. We can prove experimentally the transmission of tuberculosis from man to animals but not the converse; but children fed on the milk of scofulous cows die with tubercular meningitis.

Tubercular deposits are found in the lungs, external glands, and bone marrow, and the blood must distribute the germs to these parts. Hence if any signs of the disease are present the whole carcase is unfit for food.

*Actinomycosis* is another disease that deserves attention. Deposits once thought to be tuberculous now are seen to be of this disease. The ray fungus is easily recognized microscopically, and turns out to be very common. Actinomycosis of the bowels is known in human practice and is most probably the result of eating diseased meat. Cattle so affected are smuggled in and slaughtered and the beef sold to the poor, principally. This should be stopped.

Foot and mouth disease is a constitutional as well as a local affection, and unfits the flesh for use as food. In the "Dover outbreak" of 1884, well marked symptoms in the human subject were observed, produced by using the milk of animals sick with foot and mouth disease.

Anthrax and Trichinosis are plainly transmissible to man. But some diseases still remain, the transmissibility of which is not yet proven. Some microbes elaborate chemical poisons which produce the symptoms of disease. The disease may not be taken but the poisons are contained in the meat and make it unwholesome.

*Discussion:* Dr. Withers favors the inspection of all meat animals, both before and after slaughter; also the thorough inspection of milk and milk dairies. He favors the spaying of dairy cows as æstrum influences the quality of the milk. Dr. Williams favors strict inspection in the interest of the consumer and not of the producer. Cooking meat or boiling milk will destroy most micro-organisms but where the chief damage is done by *ptomaines;* this is not eliminated by heat.

In such cases the meat or milk should be condemned. Dr. Ryan described the inspection by Jewish officials and commended it as very thorough and highly satisfactory. The essayist spoke of a recent decision in Scotland regarding bovine tuberculosis. The *whole* carcase is now condemned if the disease is found to exist in it.

Dr. Thompson, of Paxton, then read a paper on "Fractures." He reported five cases of varying severity which recovered in slings, some with, and some without bandaging.

He always washes with the bi-chloride solution and dresses open wounds with absorbent cotton and iodoform.

*Discussion:* Dr. Williams puts some in slings and some he does not. With colts he prefers to put on a plaster bandage and turn them out Question. "How do they get up and down? Dr. Williams. "They soon learn to manage the stiffened limb." Dr. Nathers. "How do you put bandages on to stay, above the back?" Dr. Williams. "Strap with batting then with dry muslin bandages. Then stiffen the broken part with role leather splints, well washed in hot water, and cover with freshly wetted plaster bandages.

The society here adjourned until 2 P. M. On re-convening the discussion was resumed.

Dr. Withers uses slings as seldom as possible. Dr. McClintock mentioned a case of fractured humerus that was put in slings and made a good recovery. Dr. Scott has had several cases of fracture of the *os corona* and has used plaster casts and slings both with success. Dr. Withers has used the plaster casts in the cases and had good recoveries. On motion the rules were suspended and the following gentlemen proposed for membership: Drs. G. Z. Barnes (Ames '87), Pekin; and G. L. Crocker (Chicago '90), Springfield. Elected by acclamation.

Dr. Pease then read a paper on "Malaria in Horses." Three cases were reported showing more or less regular intermissions in the fever and in each case after the periodicity was noticed the above diagnosis was made and quinine was given.

It was successful in all of the cases and the essayist expressed himself in favor of large doses ʒ.1 to ʒii given so as to anticipate each paroxysm. The fever was promptly checked and convalescence progressed under a tonic as cinchonidia nux and iron. Arsenic was thought to have as good an affect as in the human subject. It proved a valuable adjuvant to the quinine, The symptoms in general were those of catarrhal fever, but without evidence of any accompanying catarrh; and the fever was intermittent and not continuous. Appetite capricious, evident headache, and later jaundice, were symptoms common.

*Discussion:* Dr. Sayre mentioned a case in his practice which was probably malaria. Dr. S. S. Baker inquired if the essayist considered ʒ.1 of quinine a large dose.

Ans.—"It proved large enough in these cases all which were in small or young animals. To a large horse I would give ʒss. and if this repeated, did not produce its desired effect would increase if necessary to ʒ1.

Dr. R. G. Walker, of Chicago, being absent, his paper was read by the secretary. He reported a singular case seen by him as well as by Dr. Baker. It appeared like a bad case of tympanitis colic, but the horse continually strained as if to pass fæces. Examination per rectum revealed a large obstruction filling the pelvic cavity. On pucturing this, per rectum, a fetid fluid was drawn off the first time, and the next time a large quantity of pus. It was washed out with a carbolic acid solution. The swelling was gone, and fæces were passed, then the horse recovered.

The discussion which was lead by Dr. S. S. Baker, who had seen the case, took up the point of the cause of this pelvic abscess. Could it have been formed in the flank by a trocar puncture and the pus have gravitated backward and downward? It was generally thought not.

Dr. Schoenleber sent in a paper on the subject of "Our College Work too Limited." It was read by the secretary and was not discussed.

Dr. Sayre then read his essay on "Veterinary Dentistry." He quoted from the oldest work on veterinary dentistry known to him, a translation from the French, by Wm. Hope, in 1596.

This writer speaks of irregularities of teeth that need attention; also of the loss of a tooth allowing the opposite one to grow into the space. From this time there was little improvement in practical dentistry until C. P. Hanse learned to examine and operate without a speculum.

Mr. Hanse though a non-graduate was a skillful operator and did much for this branch of operative surgery. The country is now full of veterinary dentist's—self-styled—many of whom do more harm than good. They have, however, drawn the attention of the public to the fact that animals suffer from their teeth ofttimes.

But we want *Veterinarians*, not *Veterinary Dentists.* There are too few operations possible to horse teeth, to make it profitable to specialize. Beside irregularities and caries, horses suffer often from Peridentitis or inflammation of the peridental membrane. It may be acute or chronic, local or general. The causes are, death of the pulp from fractures, etc., and collecting of food between the molars where it decomposes. Results alveolar abscess or exostosis on the fang. (Specimens were shown.) The fifth lower molar is most often affected. Diagnosed, by pain in mastication, and the gum receding from the tooth, and the tooth may be loose or there may be pus.

Treatment. Tr. Aconite, and Tr. Iodine equal parts applied to the gum, and a poultice to the face over the tooth.

*Discussion:* Dr. Williams drew attention to the fact that caries, does not start, as a rule, on the table surfaces of teeth. The hollow spaces in the ends of the teeth of old horses are due to the entire wearing out of the enamel at that place. Malformations occurr in which there is no dentine formed between a given two layers of enamel in the molars. This causes splitting of the teeth and caries starts from this cause. Ques. "Do the incisors ever get too long?'' Dr. Sayre.—Sometimes, and especially after much trimming of the molars, the latter do not touch without too much lateral motion of the jaws. When the mouth is in a state of repose, the molars do not touch together, in the normal mouth. Ques. "What causes chisel-shaped teeth?'' Ans. There is too much difference between the width of the upper and lower jaws.

The obliquity of the table surfaces increases until lateral motion is first retarded and then prevented. The teeth being cut off until lateral motion is possible, they improve for a time but the deformity of the jaws remains and the teeth gradually get into the same shape again.

There are cases where this is, (unilateral form as I think) partial paralysis of some of the muscles of mastication, the jaw being drawn to one side.

The committee on Constitution and By-laws reported by their chairman Dr. Williams. He read the amendments proposed and moved that they receive the sanction of the association and be laid over to the next meeting for adoption. Seconded by Dr. Withers.

After some discussion it was decided, on motion by Dr. Ryan, seconded by Dr. Scott, to amend the proposed amendment to the By-laws, by adding a clause to the code of ethics, prohibiting the offering of gratuitous advice and recipes through the veterinary columns of agricultural and other non-professional journals.

The proposed amendments, as amended, were then given the unanimous sanction of the association, and will be acted on finally at the next meeting.

In order that the members of the association might become familiar with these proposed amendment, before the November meeting, it was moved and seconded that the Constitution and By-laws, as altered by the proposed amendments, be printed in pamphlet form for distribution to all the members of the association.

It was suggested that a list of the officers' of the association from its organization to the present time, be included in this reprint together with the roll of membership. Dr. Williams included this in his motion, approved by his second. The motion was carried, and the matter of reprinting referred to the committee appointed at the last meeting for that purpose.

Thanks for the use of the hotel parlors were voted to the proprietor of the house, and Dr. Williams was appointed to convey them.

The association then adjourned to meet in Chicago in November.

J. F. PEASE,
*Recording Secretary.*

